# Myocardial perfusion quantification with Rb-82 PET: good interobserver agreement of Carimas software on global, regional, and segmental levels

**DOI:** 10.1007/s12149-022-01729-5

**Published:** 2022-02-22

**Authors:** Sergey V. Nesterov, Emmanuel Deshayes, Luis Eduardo Juarez-Orozco, Robert A. deKemp, Roberto Sciagrà, Simona Malaspina, Leonardo Settimo, Chunlei Han, Darja V. Ryzhkova, Irina S. Kostina, Kilem L. Gwet, John O. Prior, Juhani M. Knuuti

**Affiliations:** 1 Turku PET Centre, University of Turku and Turku University Hospital, Turku, Finland; 2 University of Lausanne, Lausanne, Switzerland; 3 National Cardiac PET Center, University of Ottawa Heart Institute, Ottawa, Canada; 4 University of Florence, Florence, Italy; 5 Almazov Federal Heart, Blood and Endocrinology Centre, Saint Petersburg, Russia; 6 Advanced Analytics LLC, Gaithersburg, MD USA; 7 IM Sechenov Institute of Evolutionary Physiology and Biochemistry RAS, Saint Petersburg, Russia; 8 Regional Cancer Institute of Montpellier (ICM)-Val d’Aurelle, Montpellier, France

**Keywords:** Myocardial perfusion quantification, Myocardial blood flow, Rubidium-82, Quantitative imaging, Absolute quantification, Imaging software, Agreement, Reproducibility, PET standardization, Standards in nuclear cardiology, Carimas

## Abstract

**Purpose:**

To estimate the interobserver agreement of the Carimas software package (SP) on global, regional, and segmental levels for the most widely used myocardial perfusion PET tracer—Rb-82.

**Materials and methods:**

Rest and stress Rb-82 PET scans of 48 patients with suspected or known coronary artery disease (CAD) were analyzed in four centers using the Carimas SP.

We considered values to agree if they simultaneously had an intraclass correlation coefficient (ICC) > 0.75 and a difference < 20% of the median across all observers.

**Results:**

The median values on the segmental level were 1.08 mL/min/g for rest myocardial blood flow (MBF), 2.24 mL/min/g for stress MBF, and 2.17 for myocardial flow reserve (MFR). For the rest MBF and MFR, all the values at all the levels fulfilled were in excellent agreement. For stress MBF, at the global and regional levels, all the 24 comparisons showed excellent agreement. Only 1 out of 102 segmental comparisons (seg. 14) was over the adequate agreement limit—23.5% of the median value (ICC = 0.95).

**Conclusion:**

Interobserver agreement for Rb-82 PET myocardial perfusion quantification analyzed with Carimas is good at any LV segmentation level—global, regional, and segmental. It is good for all the estimates—rest MBF, stress MBF, and MFR.

## Introduction

Myocardial perfusion imaging with PET enables quantification of the myocardial blood flow (MBF) in absolute terms (mL/min/g). The three clinically available PET perfusion tracers are O-15 water, N-13 ammonia, and Rb-82—the latter being the most widely used [1], likely because its production does not require an on-site cyclotron. Also, Rb-82 has been extensively validated for clinical practice [2].

A fundamental part of myocardial perfusion quantification (MPQ) with PET is the image's software-based transformation into MBF estimates. This transformation completes through tracer kinetic modeling (TKM), which fits a tissue compartment model (TCM) to the data—the registered radioactive counts through the scan time. Although quite a few TCMs have been published and validated, our cross-comparison study of those published and implemented models for Rb-82 demonstrated that the differences between the values received with different models could be as high as 130% [3].

The lack of confidence in MBF values provided by dedicated software packages has gradually come to the forefront [4–6]. One of the critical elements of such trust is the SP's internal reproducibility—the agreement between repeat analyses done by different observers using the same software package (SP)—i.e., users of an SP must get results that would agree [7–9].

This study focused on analyzing the interobserver agreement of Carimas SP on all the levels—global, regional, and segmental—for the most widely used tracer—Rb-82.

## Materials and methods

All Rb-82 PET studies were performed at the Department of Nuclear Medicine of the University Hospital of Lausanne (Switzerland) according to routine clinical practice. The local ethics committee approved the study protocol. Each patient provided written informed consent before the study.

Forty-eight (*N* = 48) consecutive patients with suspected or known CAD were studied after an overnight fast and were instructed to forego caffeine- or theophylline-containing products or medications 24 h before the study. The patients underwent rest and adenosine-induced stress Rb-82 PET.

### PET image acquisition

A brief CT scout was acquired, followed by a CT attenuation correction (AC) scan (120 kV, 10 mA); CT AC image alignment with PET was verified visually by an experienced technologist and corrected, if necessary, by the manual 3D translation, using the vendor's program.

PET scans were acquired on a Discovery 690 PET/CT (GE Healthcare, Milwaukee, WI, USA) using a 3D list-mode acquisition after a 30-s (constant-activity square-wave) infusion of Rb-82 (10 MBq/kg, Jubilant DraxImage, Kirkland, Canada). An 8-min rest acquisition was started ~ 10–15 s after starting the intravenous Rb-82 infusion. Following the rest data acquisition, patients underwent a pharmacological stress study. The patient kept the same position, while adenosine (0.84 mg/kg) was infused over 6 min. Two minutes after the start of adenosine infusion, Rb-82 infusion was started. The PET acquisition for pharmacological stress was performed the same way as described for the rest [10].

Dynamic images were reconstructed using the vendor VPFX time-of-flight algorithm (two iterations and 24 subsets) into 24 time frames (12 × 8 s, 5 × 12 s, 1 × 30 s, 1 × 60 s, 2 × 120 s), with 6.4 mm 3D Gaussian post-filtering.

### PET image analysis

The reconstructed images—rest and stress—were delivered to four PET facilities in Finland, Italy, Russia, and Switzerland. The four investigators utilized the Carimas SP [3, 7–9] and were blind to the other readers' results.

#### Carimas

In Carimas (Turku PET Centre, University of Turku and Turku University Hospital, Turku, Finland), the myocardial segmentation is done semi-automatically—a user defines long and short axes. The input function from the LV cavity is defined as a reduced volume of the LV.

Carimas has two ways to control and assure the image analysis quality: (a) visualization of segmentation results with broad manual adjusting capabilities and (b) plotting the fitted TAC with corresponding data and goodness of fit displayed in the modeling results table.

**Fig. 1:**
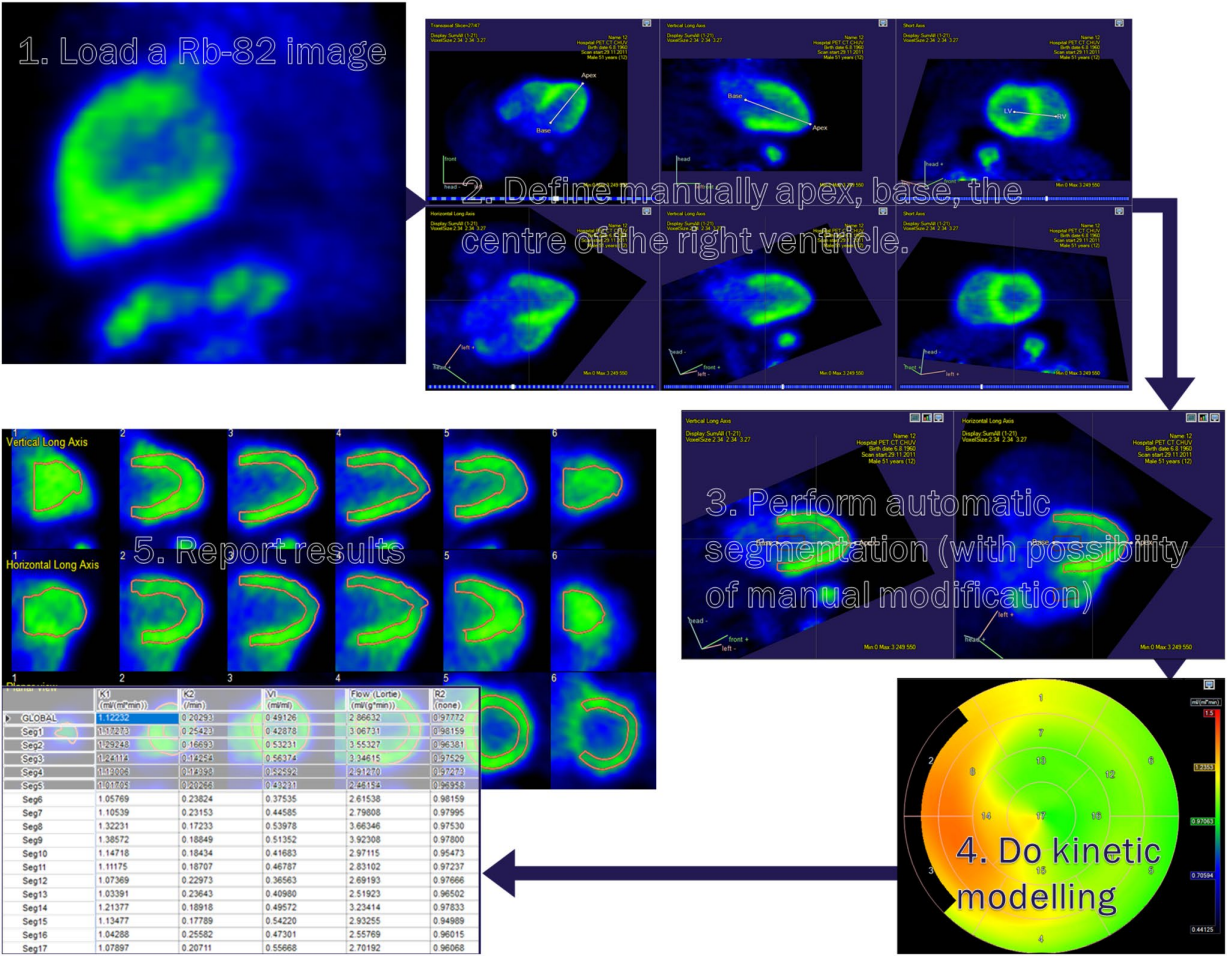
The analysis workflow in Carimas (performed for an Rb-82 stress MBF image)

For Rb-82 PET, Carimas implemented the one-tissue compartment model (1TCM) suggested by Lortie et al. in 2007 [11]. The program calculates global MBF based on a myocardial global TAC; the same principle applies to regional and segmental—17-segment AHA model [12]—MBF values. Figure 1 shows the analysis workflow in Carimas using an Rb-82 stress MBF image as an example.

#### The studied values

Image analysis delivered global, regional, and segmental values for three parameters: rest MBF, stress MBF, and myocardial flow reserve (MFR), which is the unitless ratio of the stress to the rest MBF values. Global values were averaged over the whole LV; regional values considered the three vascular territories (left anterior descending [LAD], left circumflex [LCx], and right coronary artery [RCA]), and the segmental values followed the established 17-segment AHA standard model [12].

The four observers provided six comparisons for any studied parameter (the formula is *N*(*N *− 1)/2). Thus, we had six comparisons for each global flow parameter—six for rest MBF, six for stress MBF, and six for MFR (e.g., *Observer1 Global MFR* vs. *Observer4 Global MFR*). We had 18 comparisons (six by three) for each flow parameter for the three vascular territories—e.g., *Observer2 RCA sMBF* vs. *Observer3 RCA sMBF*. We had 102 comparisons (6 × 17) for each parameter for the segments—e.g., *Observer3 seg10 rMBF* vs. *Observer4 seg10 rMBF*.

### Statistical analysis

The number of compared sets (four) exceeded two, and we could not use the standard approach to measure the agreement proposed by Bland and Altman [13]; therefore, we applied a custom linear mixed model for repeated measures (MMRM) [14]. The statistical model output included two main agreement metrics—intraclass correlation coefficient (ICC) and the absolute difference between values from the implemented TKM—both calculated for each comparison.

We considered the agreement between models sufficient if the absolute difference was less than 20% of the corresponding median across all observers and the corresponding ICC ≥ 0.75. We introduced this criterion for the acceptable discrepancy in our earlier work [2], accounting for previous reports in the field [15] and the reported variability of within-subject perfusion estimates [16], and based the standard for the adequate ICC threshold on the literature data [17]. [Of note, the 20% difference can be seen as substantial (the concerns for its magnitude are in [18]), yet, when there are no other generally accepted cutoff values of the sort, it serves us a decent seamark to navigate safely.]

We expressed the difference between MBF and MFR values as a percent of their corresponding medians to unify the scale of disagreements through all the estimated parameters.

### Biplot visualization

To visualize a large number of results, we used a custom biplot [3], relating the two defined metrics—the differences and the ICC values of compared pairs. In this plot, the *X* axis shows pairwise differences between the model values, and the *Y* axis shows corresponding pairwise values of 1 − ICC. In this biplot, the origin (*x* = 0 and *y* = 0) is the point of identity between the compared values, where there is no absolute difference, and the ICC is equal to 1. Thus, the farther the values are from the origin, the less they agree—either showing an absolute difference or a reduced ICC. The predefined criteria for the good agreement were a rectangular region on the biplot. Thus, this biplot intuitively visualizes our agreement criteria—the dots inside these borders represented pairs with the high agreement.

## Results

### Patient characteristics and hemodynamics

The study population demographics and hemodynamic characteristics are in Table 1.

**Table 1:** Population characteristics

Number of subjects	48
Number of males (% of total)	35 (73%)
Age, years. (range)	63 ± 12.7 (33–87)
Weight, kg (range)	79 ± 15.3 (48–116)
Body mass index, kg/m^2^ (range)	27.0 ± 4.78 (16.0–41.7)
Symptoms	36 (75%)
Angina	28 (58%)
Dyspnoea	27 (56%)
Family history of cardiovascular disease	14 (29%)
Known CAD	24 (50%)
Previous myocardial infarction	15(31%)
Received procedures	20 (42%)
Coronary artery bypass graft surgery	5 (10%)
Percutaneous coronary intervention	17 (35%)
Hypercholesterolaemia	29 (60%)
Arterial hypertension	38 (79%)
Diabetes mellitus	10 (21%)
Currently smoking or ex-smoker	28 (58%)
Hemodynamics at rest	
Heart rate, beats/min (range)	76 ± 17.0 (49–135)
Systolic blood pressure, mm Hg (range)	136 ± 22.3 (94–212)
Diastolic blood pressure, mm Hg (range)	71 ± 13.3 (46–110)
Rate pressure product, mm/min (range)	10,400 ± 2870 (6000–18,900)
Hemodynamics at pharmacological stress	
Heart rate, beats/min (range)	85 ± 15.6* (48–135)
Systolic blood pressure, mm Hg (range)	131 ± 21.1^†^ (70–183)
Diastolic blood pressure, mm Hg (range)	68 ± 15.1^†^ (30–115)
Rate pressure product, mm/min (range)	11,200 ± 2870^‡^ (6100–21,600)

Values are *n* (%) or arithmetic mean ± SD

*p < 0.001 vs. rest; ^†^*p* < 0.05 vs. rest; ^‡^*p* < 0.01

As expected, during the pharmacological stress test, the heart rate increased (*P* < 0.001). At the same time, blood pressure showed a mild decrease (*P* < 0.05), resulting in a rate pressure product (RPP) net increase of approximately 7.7% (*P* < 0.01). All 48 patients, including the one with a blood pressure of 70/30 mm Hg during stress, tolerated the pharmacological stress test well.

### Measurement of MBF and MFR in absolute values

The obtained absolute perfusion values are in Table 2. The median values on the segmental level were 1.08 mL/min/g for rest MBF, 2.24 mL/min/g for stress MBF, and 2.17 for MFR.

**Table 2:** MBF and MFR values

	Rest MBF				Stress MBF				MFR			
	OBS_1	OBS_2	OBS_3	OBS_4	OBS_1	OBS_2	OBS_3	OBS_4	OBS_1	OBS_2	OBS_3	OBS_4
Global	1.11 ± 0.36	1.12 ± 0.39	1.12 ± 0.36	1.11 ± 0.36	2.38 ± 1.04	2.44 ± 1.12	2.48 ± 1.05	2.38 ± 1.07	2.18 ± 0.82	2.25 ± 0.92	2.27 ± 0.86	2.19 ± 0.85
LAD	1.12 ± 0.38	1.13 ± 0.41	1.14 ± 0.38	1.12 ± 0.38	2.31 ± 1.09	2.39 ± 1.16	2.51 ± 1.15	2.32 ± 1.12	2.10 ± 0.87	2.18 ± 0.94	2.25 ± 0.89	2.12 ± 0.88
LCx	1.08 ± 0.33	1.09 ± 0.34	1.09 ± 0.32	1.09 ± 0.33	2.30 ± 0.92	2.31 ± 0.96	2.30 ± 0.88	2.27 ± 0.93	2.17 ± 0.75	2.19 ± 0.81	2.15 ± 0.75	2.14 ± 0.73
RCA	1.16 ± 0.42	1.15 ± 0.43	1.11 ± 0.41	1.13 ± 0.42	2.86 ± 1.32	2.86 ± 1.42	2.82 ± 1.38	2.80 ± 1.38	2.54 ± 1.07	2.58 ± 1.16	2.58 ± 1.13	2.55 ± 1.12
Seg01	1.07 ± 0.31	1.07 ± 0.32	1.05 ± 0.32	1.05 ± 0.32	2.06 ± 0.65	2.10 ± 0.71	2.06 ± 0.64	2.07 ± 0.70	2.04 ± 0.72	2.09 ± 0.81	2.14 ± 0.90	2.10 ± 0.77
Seg02	1.11 ± 0.33	1.12 ± 0.35	1.10 ± 0.34	1.10 ± 0.34	2.34 ± 0.74	2.41 ± 0.87	2.36 ± 0.78	2.37 ± 0.85	2.21 ± 0.72	2.25 ± 0.76	2.29 ± 0.83	2.24 ± 0.73
Seg03	1.21 ± 0.41	1.18 ± 0.41	1.12 ± 0.40	1.17 ± 0.41	2.92 ± 1.16	2.90 ± 1.32	2.76 ± 1.26	2.84 ± 1.26	2.54 ± 0.96	2.54 ± 0.99	2.54 ± 1.07	2.54 ± 1.01
Seg04	0.97 ± 0.36	0.97 ± 0.36	0.90 ± 0.34	0.95 ± 0.37	2.40 ± 1.10	2.35 ± 1.17	2.17 ± 1.28	2.37 ± 1.19	2.61 ± 1.17	2.53 ± 1.17	2.39 ± 1.17	2.59 ± 1.21
Seg05	0.96 ± 0.31	0.97 ± 0.32	0.94 ± 0.30	0.97 ± 0.31	2.14 ± 0.94	2.11 ± 0.95	1.99 ± 0.87	2.15 ± 0.97	2.28 ± 0.88	2.22 ± 0.88	2.13 ± 0.83	2.26 ± 0.86
Seg06	1.06 ± 0.31	1.10 ± 0.32	1.06 ± 0.30	1.07 ± 0.31	2.08 ± 0.76	2.12 ± 0.77	2.05 ± 0.70	2.10 ± 0.79	2.01 ± 0.66	2.03 ± 0.73	2.02 ± 0.72	2.02 ± 0.67
Seg07	1.24 ± 0.37	1.22 ± 0.41	1.26 ± 0.36	1.23 ± 0.38	2.39 ± 0.86	2.40 ± 0.95	2.51 ± 0.89	2.40 ± 0.89	2.03 ± 0.79	2.08 ± 0.84	2.11 ± 0.80	2.05 ± 0.77
Seg08	1.31 ± 0.44	1.33 ± 0.48	1.34 ± 0.42	1.32 ± 0.44	2.72 ± 1.08	2.87 ± 1.20	3.03 ± 1.19	2.85 ± 1.17	2.17 ± 0.83	2.26 ± 0.85	2.35 ± 0.85	2.23 ± 0.79
Seg09	1.35 ± 0.48	1.32 ± 0.49	1.28 ± 0.45	1.31 ± 0.47	3.20 ± 1.34	3.28 ± 1.49	3.28 ± 1.46	3.18 ± 1.42	2.46 ± 0.95	2.58 ± 1.08	2.65 ± 1.08	2.52 ± 1.03
Seg10	1.17 ± 0.44	1.15 ± 0.46	1.12 ± 0.43	1.13 ± 0.43	2.99 ± 1.44	2.93 ± 1.51	2.84 ± 1.39	2.85 ± 1.46	2.67 ± 1.24	2.69 ± 1.30	2.61 ± 1.18	2.63 ± 1.25
Seg11	1.11 ± 0.37	1.11 ± 0.38	1.11 ± 0.36	1.11 ± 0.37	2.59 ± 1.17	2.56 ± 1.19	2.54 ± 1.12	2.48 ± 1.11	2.42 ± 1.05	2.39 ± 1.06	2.32 ± 0.91	2.32 ± 0.98
Seg12	1.19 ± 0.35	1.20 ± 0.38	1.22 ± 0.34	1.20 ± 0.35	2.42 ± 0.93	2.42 ± 0.97	2.46 ± 0.90	2.39 ± 0.92	2.09 ± 0.70	2.11 ± 0.77	2.08 ± 0.69	2.05 ± 0.66
Seg13	1.11 ± 0.37	1.11 ± 0.42	1.16 ± 0.37	1.13 ± 0.38	2.21 ± 1.00	2.26 ± 1.10	2.37 ± 1.05	2.23 ± 1.05	2.05 ± 0.91	2.11 ± 0.99	2.12 ± 0.97	2.05 ± 0.96
Seg14	1.25 ± 0.52	1.29 ± 0.58	1.32 ± 0.50	1.28 ± 0.51	2.65 ± 1.44	2.86 ± 1.53	3.17 ± 1.62	2.78 ± 1.45	2.18 ± 1.01	2.31 ± 1.11	2.45 ± 1.08	2.25 ± 1.00
Seg15	1.21 ± 0.49	1.21 ± 0.53	1.19 ± 0.47	1.17 ± 0.46	3.00 ± 1.91	3.07 ± 1.88	3.19 ± 1.89	2.90 ± 1.70	2.52 ± 1.48	2.67 ± 1.70	2.75 ± 1.47	2.54 ± 1.42
Seg16	1.12 ± 0.37	1.11 ± 0.39	1.15 ± 0.36	1.12 ± 0.37	2.41 ± 1.17	2.42 ± 1.16	2.53 ± 1.11	2.33 ± 1.09	2.21 ± 0.98	2.26 ± 1.01	2.26 ± 0.93	2.14 ± 0.89
Seg17	1.09 ± 0.43	1.13 ± 0.52	1.10 ± 0.44	1.08 ± 0.40	2.34 ± 1.38	2.41 ± 1.41	2.53 ± 1.42	2.26 ± 1.28	2.18 ± 1.05	2.24 ± 1.17	2.32 ± 1.01	2.13 ± 1.01

Values are means ± SD (n = 48)

### Interobserver agreement of the myocardial perfusion estimates

**Fig. 2:**
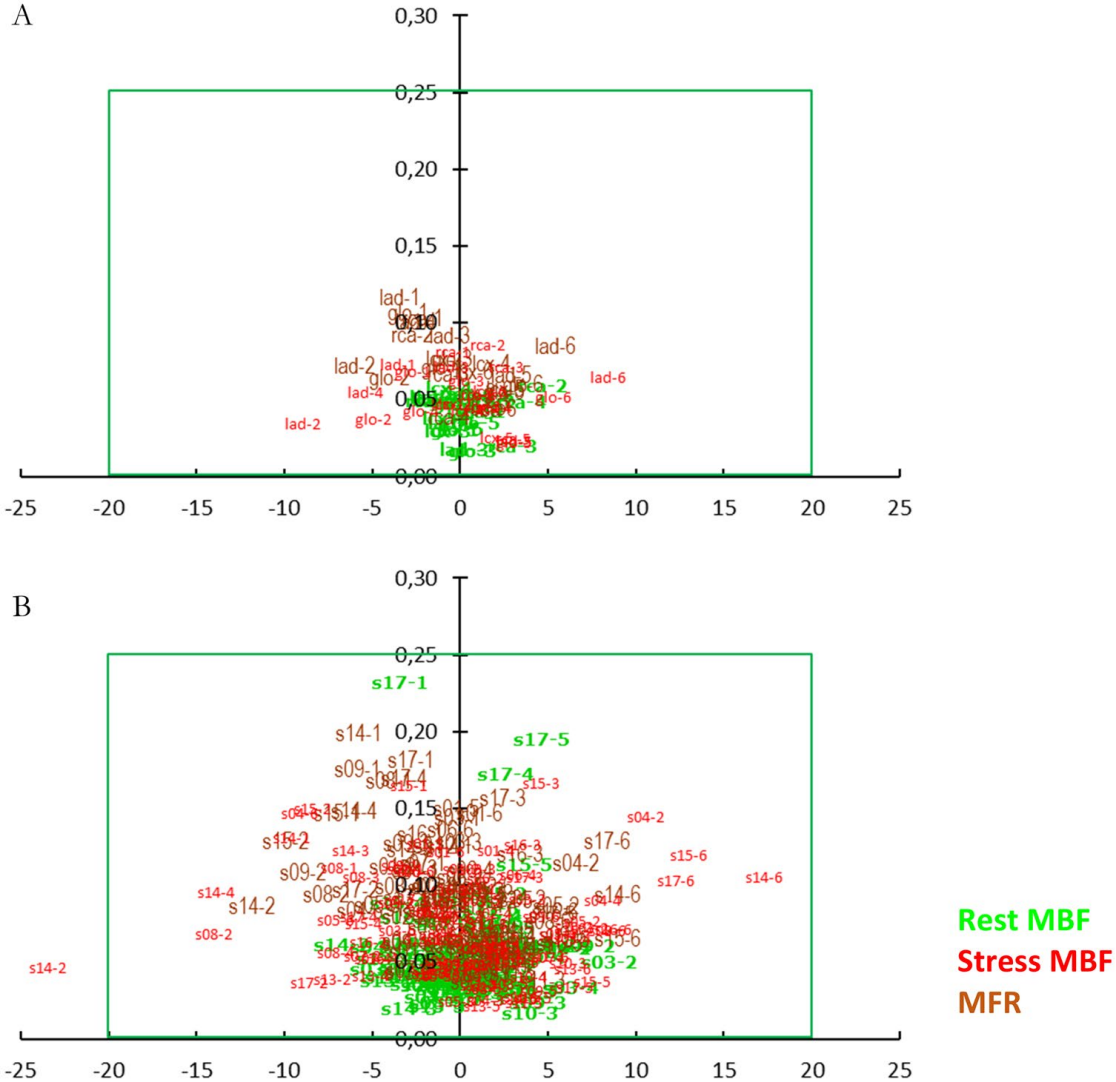
Cross-comparison biplot for MBF and MFR values: **A** on global and regional levels, **B** on the segmental level. The *X* axis is the difference in MBF and MFR values presented as a percentage of the corresponding median; the *Y* axis is 1-ICC. The *x* range of the green outline represents ± 20% of the median value for Rb-82 cross-comparisons; the *y* range—ICC values over 0.75. The green rectangle encompasses the area of excellent agreement. The chart element is the compared area (e.g., s14) and an ordinal number of the six comparisons from 1 to 6

The agreement values are in Fig. 2: A—for global and regional levels, B—for the segmental level. For the rest MBF and MFR, all the values at all the levels—global, regional, segmental—fulfilled both the requirements for the excellent agreement. Moreover, for the rest MBF, 90% of differences did not exceed 3% of the corresponding median on the global and regional levels and 4.5% at the segmental level. For MFR, 90% of the differences did not exceed 5% of the corresponding median at the global and regional levels and 6% at the segmental level. The most considerable difference was 11.8% of the median (ICC = 0.91) for MFR in segment 14 (distal septal).

For stress MBF, at the global and regional levels, all the 24 comparisons showed excellent agreement. It was the case that 90% of differences did not exceed 6% of the corresponding median, while at the segmental level, 90% of the differences did not exceed 9%. Only 1 out of 102 comparisons, again in segment 14, was over the adequate agreement limit—23.5% of the median value. Still, the ICC was 0.95.

### Discussion

We evaluated the interobserver agreement of PET MPQ for the most widely used tracer—Rb-82—using Carimas SP. Different operators analyzed the data from 48 scanned patients with suspected or known CAD in four centers. The observers had different levels of experience with the software—from an observer who learned Carimas to analyze these data to observers routinely using it. Notwithstanding, the results were highly reproducible at every level of analysis—global, regional, and segmental—for all the obtained perfusion estimates (rest MBF, stress MBF, and MFR).

Our study's particular feature was the level range achieved by evaluating perfusion estimates' reproducibility at all segmentation levels. Researchers who have cross-compared myocardial PET SPs avoided the segmental level, except for our group's studies using O-15 water [7] and C-11 acetate [8]. However, averaging multiple segments in a single vascular territory underestimates visually discernible perfusion defects, making segmental analysis clinically relevant. Therefore, to envision the “clinical reality” of MPQ, we cannot bypass this level—it simply provides us with more information on the myocardium. Also, as shown by Berti et al. [19], the assessment of absolute myocardial perfusion parameters measured at a segment level leads to reliable and accurate identification of patients with significant coronary stenosis at invasive coronary angiography (ICA) and/or coronary computed tomography angiography (CCTA).

In this study, the interobserver differences for MBF and MFR values were less than the predefined 20%: the majority (90%) were < 4.5% for segmental rest MBF, < 9% for segmental stress MBF, and < 6% for segmental MFR. These differences were smaller than the corresponding segmental differences reported previously in the RUBY-10 project [3] (90% of those differences < 25% for segmental rest MBF, 32% for segmental stress MBF, and 16.5% for segmental MFR), which utilized the same patient set and also implemented the 1TCM [11] to investigate the agreement across eight different SPs. Therefore, this study's excellent agreement might originate from the uniformity of observer-independent processes in the Carimas SP, such as image reorientation or segmentation.

We know that SPs vary in operator interaction and manipulation necessary for the adequate reconstruction and, therefore, for reliable MPQ. Some SPs like QPET and Syngo MBF require minimal observer interaction; others like PMOD and Carimas assume more manual adjustment in the alignment and border detection of the observer to provide accurate MPQ. There hardly can be a winner here yet. Although the current capabilities of machines allow for the complete automatization of many processes and results would likely be congruent, such automatization of the analysis process would necessarily pose a new question: who will be liable for the diagnosis? Machines cannot be, so human observers will want a possibility to interfere with the analysis process. And by doing so, they will bring their sources of possible discrepancies.

Further research into the interobserver reproducibility of MPQ across different SPs might be enlightening. However, we can already speculate that intra-SP reproducibility will generally be higher than between SPs (considering our results and the results from the RUBY-10 together).

Currently, we can suggest that if clinical analysts use Carimas for Rb-82 PET MPQ, they can safely assume that their results will be reproducible.

### Limitations

The limitation of this study is the absence of a gold standard, so we cannot claim, in this paper, the quantitative accuracy of Carimas SP. Yet, the study's goal was to assess the reproducibility and not accuracy regarding absolute values.

We did not study the software agreement as a function of types of CAD or degrees of its severity. We reflected this limitation in the title, and the paper itself, calling the agreement we received “good” and not “excellent.” However, technically, the resulting level of agreement could have been termed "excellent."

We used only Rb-82 data from one center, acquired on one scanner, reconstructed with one algorithm. We do not consider it a limitation, because introducing these new variables into our combinatorial study would have led to the project's practical impossibility.

We treat the imminent need for standardization in PET MPQ in several papers [4, 6, 20].

## New knowledge gained

Interobserver agreement on the segmental level can be good for Rb-82 PET MPQ.

## Conclusions

Interobserver agreement for Rb-82 PET myocardial perfusion quantification analyzed with Carimas is good at any LV segmentation level—global, regional, and segmental. It is good for all the estimates—rest MBF, stress MBF, and MFR.

## Data Availability

In CHUV, Lausanne, Switzerland.

## Abbreviations

ICC: Intraclass correlation coefficient
LV: Left ventricle
MBF: Myocardial blood flow
MFR: Myocardial flow reserve
MMRM: Linear mixed model for repeated measures
MPQ: Myocardial perfusion quantification
SP: Software package
TCM: Tissue compartment model
TKM: Tracer kinetic modeling

## Declarations

### Code availability

Carimas is not an open-source software.
